# Metal-Free Organic Radical Spin Source

**DOI:** 10.1021/acs.nanolett.3c01044

**Published:** 2023-05-08

**Authors:** Constantinos Nicolaides, Fadwat Bazzi, Evangelos Vouros, Dragos F. Flesariu, Nicolas Chrysochos, Panayiotis A. Koutentis, Christos P. Constantinides, Theodossis Trypiniotis

**Affiliations:** † Department of Physics, University of Cyprus, P.O. Box 20537, 1678 Nicosia, Cyprus; ‡ Department of Natural Sciences, University of Michigan − Dearborn, 4901 Evergreen Rd, Dearborn, Michigan 48128-1491, United States; § Department of Chemistry, University of Cyprus, P.O. Box 20537, 1678 Nicosia, Cyprus

**Keywords:** Organic spintronics, Organic radical, Spin pumping

## Abstract

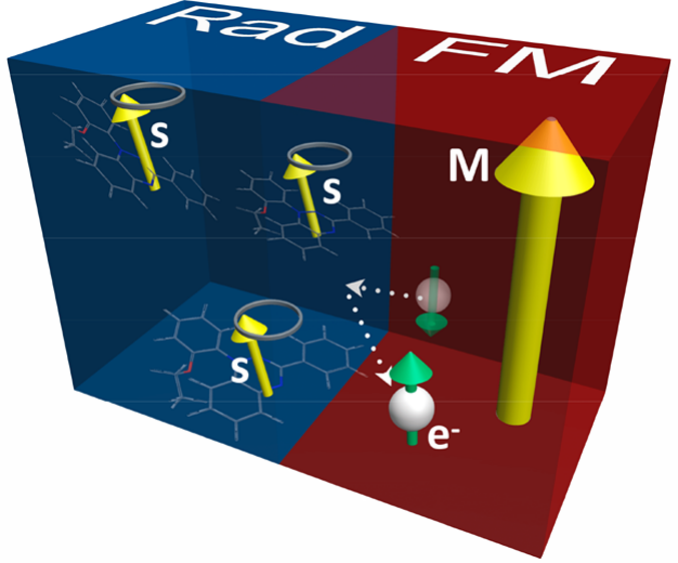

Organic radicals have long been suggested as candidates for organic magnets and components in organic spintronic devices. Herein, we demonstrate spin current emission from an organic radical film via spin pumping at room temperature. We present the synthesis and the thin film preparation of a Blatter-type radical with outstanding stability and low roughness. These features enable the fabrication of a radical/ferromagnet bilayer, in which the spin current emission from the organic radical layer can be reversibly reduced when the ferromagnetic film is brought into simultaneous resonance with the radical. The results provide an experimental demonstration of a metal-free organic radical layer operating as a spin source, opening a new avenue for the development of purely organic spintronic devices and bridging the gap between potential and real applications.

Organic spintronic device concepts and many of the studies reported to date rely on inorganic ferromagnets as an emitter or detector of spin.^1−4^ A major shift in the field of organic spintronics will take place if the ferromagnets (FMs) can be replaced by metal-free organic equivalents, since organic synthesis can enable easy spintronic device design at the molecular level, e.g., via integration with semiconducting polymers. The search for such organic magnetic materials at room temperature has been the focus of various investigations,^5−7^ and recently, the existence of room temperature ferromagnetism in oxidized perylene diimide powder was reported.^8^

The ability to create pure spin currents via spin pumping^9^ from a paramagnet was recently reported, demonstrating that long-range magnetic order is not a prerequisite for a spin emitter material.^10^ The double-perovskite oxide paramagnetic insulator, La_2_NiMnO_6_, provides paramagnetic spin pumping of a pure spin current at room temperature with comparable efficiency to that of typical spin pumping devices involving ferromagnets.^10^ Even though paramagnets are relatively understudied spintronic materials, initial studies show that they can support spin current injection, transport,^11−13^ and the spin Seebeck effect,^14,15^ setting the basis for future paramagnetic spintronic applications.

The use of an organic paramagnet as a spin current source is a promising route to achieve a fully organic spintronic device. Stable organic radicals, a class of molecules containing one or more unpaired electrons, are an obvious choice for such a paramagnet. Since every radical molecule contains at least one free electron, a solid-state radical layer ensures high spin concentration. The shortest intermolecular interactions between organic radicals often offer the pathways to propagate the magnetic exchange interactions between the unpaired electrons located on the SOMOs of the nearest neighbor molecules. This could be the cause of short-range magnetic correlation in radical layers.^5^ A particular class of organic radicals, 1,2,4-benzotriazinyls, named after Blatter who first reported them,^16^ has remarkable stability in ambient conditions but was underexplored owing to limited availability. Recent improvements in the synthesis of Blatter radicals^17−20^ enabled access to structurally diverse analogues tailored to different applications and increased the general interest for these molecules.^21^ For example, a pyrene-Blatter type radical derivative has been proposed as a potential quantum bit,^22^ while a polymeric Blatter radical was investigated as a cathode material in organic batteries.^23^ Moreover, a F_3_C substituent Blatter type radical showed an important Seebeck enhancement in comparison to an analogous closed-shell molecule, making it a candidate organic thermoelectric material,^24^ while a high-spin diradical, comprised of two Blatter-type radical moieties, displayed electrical conductivity and remarkable metal-like behavior at low temperatures.^25^

The nature of the unpaired electron combined with Blatter-type radicals’ inherent stability makes them ideal candidates on which to develop spintronics, and recently, their use as building blocks for organic magnets was widely suggested.^21,26^ It would be beneficial, for both spintronic studies and future devices, to be able to fabricate robust thin radical layers, ideally in contact with other materials with known properties, e.g., metallic films, without alteration of the radical characteristics. The stability of Blatter-type radical monolayers or thin layers under ambient conditions is still a challenge and is the focus of several studies^27,28^ with some initial steps toward successful fabrication of radical thin layers with robust stability already realized.^25,28^ Of particular importance to spintronics is the possibility of engineering Blatter-type radical materials with strong magnetic exchange interactions to reveal, and subsequently harness, the correlation between structure and magnetism in these systems.^29−32^

Despite the recent rapid progress in this field, the question of whether a radical can act as a spin source in an organic spintronic device remains unanswered. In this study, we show that such a radical film can fulfill the role of a source of spin current in a way that is analogous to spin pumping from a ferromagnet. We fabricate a stable Blatter-type radical/NiFe bilayer and carry out simultaneous electron spin resonance (ESR) and ferromagnetic resonance (FMR) measurements. A signature of spin current emission from the radical is obtained through its increased linewidth. By carefully tuning the two resonances to coincide, we demonstrate that the radical linewidth increase can be reversibly reduced due to emission of a backward spin current from the FM layer, effectively canceling the radical’s emission. This successful use of a radical film as a spin current source indicates the potential of radicals as an alternative to conventional metallic ferromagnets in spintronics.

We carefully selected a Blatter-type radical, 1-(2-ethoxyphenyl)-3-phenyl-1,4-dihydro-1,2,4-benzotriazin-4-yl (EBR), to enable easy formation of thin films. In a recent study, we reported a 1-(2-methoxyphenyl) (MBR) equivalent radical (Figure 1a), the orthogonal structure of which suppresses the known propensity of the radical to crystallize in 1D columns.^30^ In this study, to encourage the formation of uniform thin films, we introduced a subtle structural modification by exchanging the 1-(2-methoxyphenyl) with a more lipophilic and solubilizing 1-(2-ethoxyphenyl) substituent. We synthesized the 1-(2-ethoxyphenyl)-substituted Blatter radical (Figure 1a) via the procedure detailed in the Supporting Information (SI). The 2-ethoxyphenyl Blatter-type radical thin film was fabricated by spin coating a toluene solution of EBR (5 mg/mL) on a Si/SiO_x_ substrate at 2000 rpm in ambient conditions. A uniform radical film was obtained with a noteworthily low roughness, 250 ± 30 pm, measured by atomic force microscopy (AFM) (details in the SI), which is at least 1 order of magnitude lower compared to a previous report.^28^ The ESR spectrum of the EBR in solution (Figure 1b) shows hyperfine coupling of the unpaired electron with three neighboring nitrogen nuclei, with a measured *g*-factor of 2.0040 (see SI). The ESR spectra of the solution and the thin film are presented in Figure 1b. The *g*-factor remains unaffected in the thin film and the hyperfine interaction splitting pattern disappears as expected in the solid state,^33,34^ since at high spin concentration the spin–spin exchange dominates the hyperfine interaction. The ESR spectra of the film resemble a single Lorentzian function (see SI), demonstrating that the EBR molecules in the film behave as a homogeneous broadening system^35^ without any inhomogeneous contribution arising by their possible alteration at the interface. The EBR film was subsequently monitored by ESR spectroscopy over a period of one month in ambient conditions at room temperature. Figure 1c shows the normalized ESR intensity as a function of time in logarithmic scale from day 0 to day 30, which practically remains constant, indicating outstanding stability under ambient conditions.

**Figure 1:**
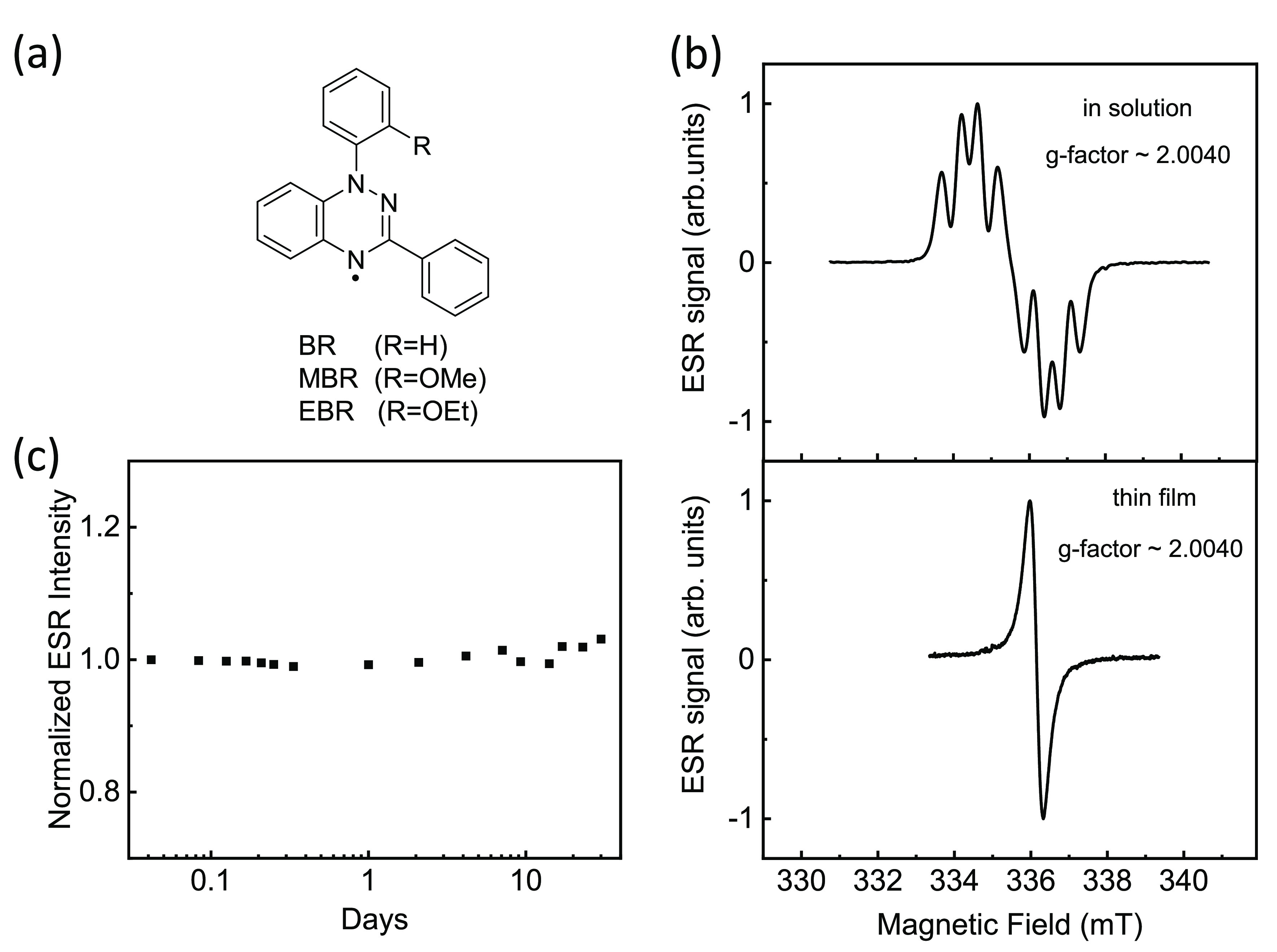
(a) Structures of the parent Blatter radical (BR) along with the 1-(2-methoxyphenyl) (MBR) and the 1-(2-ethoxyphenyl) (EBR) analogues. (b) Electron spin resonance (ESR) spectrum of the EBR in solution and in a thin film form with application of the magnetic field in the film plane. (c) Normalized ESR intensity as a function of time for a period of one month.

Following the creation of a stable Blatter-type radical film with low roughness, we fabricated a Blatter-type radical film/ferromagnet bilayer to investigate spin current transport and spin interactions through the interface between the two layers. A NiFe ferromagnetic thin film (7 nm) was deposited onto a Si/SiO_x_ substrate by molecular beam epitaxy (see SI for details) followed by a spin coated EBR thin layer of ∼25 nm (measured by AFM). This bilayer as well as the previous ESR samples were placed at the center of a TE_102_ rectangular microwave cavity with an operational frequency of 9.43 GHz, in which the angle *θ*_H_ of the external magnetic field with respect to the sample normal can be controlled. Figure 2a presents typical ESR/FMR spectra of the EBR/NiFe bilayer at *θ*_H_ = 0° (external applied magnetic field in the sample’s plane). The observation of FMR and ESR spectra indicates the successful deposition of the EBR onto the NiFe layer without alteration of the spin or magnetic dynamics, respectively.^36^ This is further confirmed by comparing the *g*-factor of the radical for all three sample cases, in solution, as a single layer and as a bilayer, which have approximately the same *g ≈* 2.004 (Figures 1b and 3b). Similarly, the ESR spectrum is a single Lorentzian distribution indicating that the EBR film still behaves as a homogeneous system (see SI).

**Figure 2:**
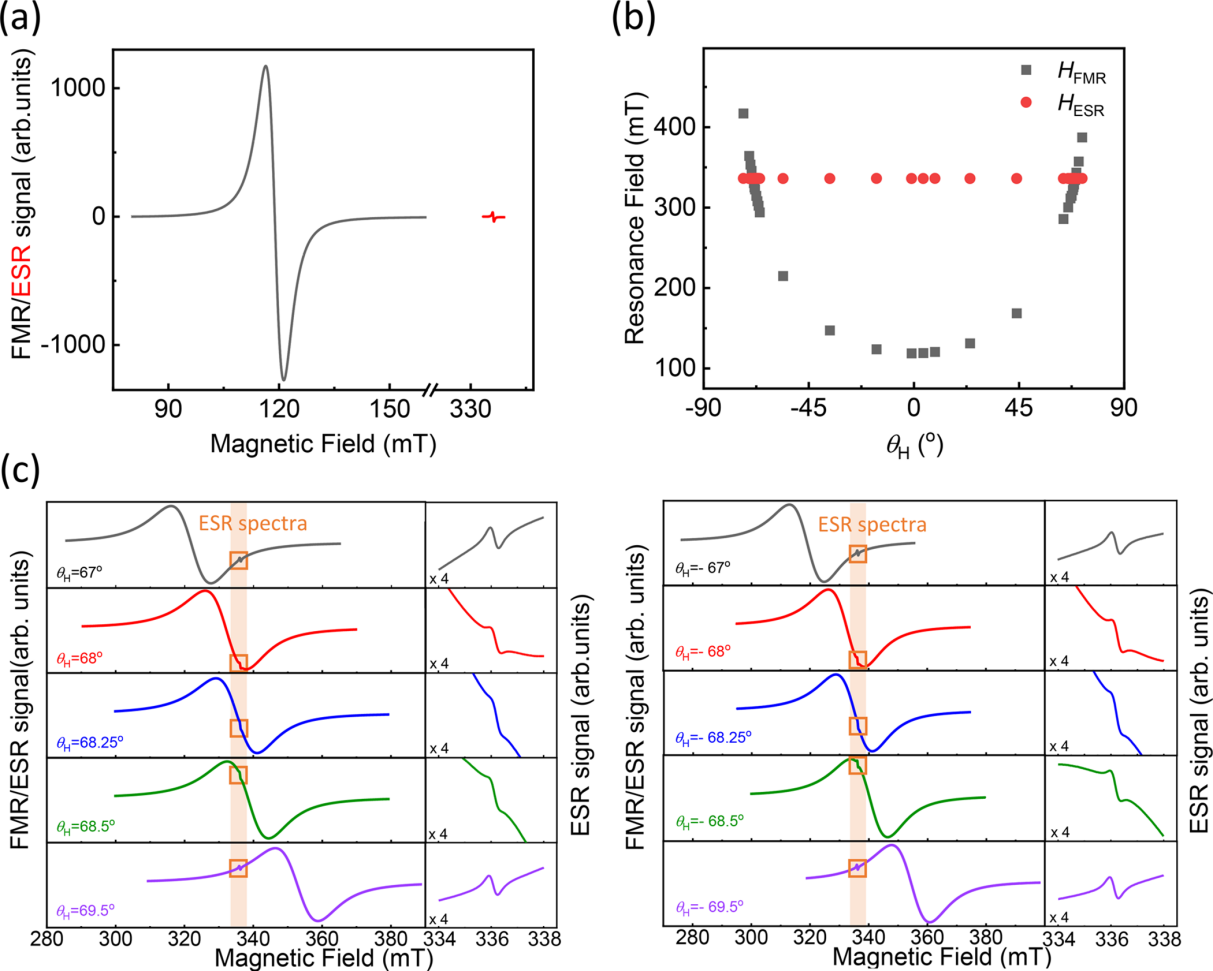
(a) Typical ESR/FMR spectra for an EBR/NiFe bilayer for *θ*_H_ = 0°. (b) The magnetic field angle, *θ*_H_, dependence of the resonance fields *H*_FMR_ and *H*_ESR_ for the EBR/NiFe bilayer. (c) Detailed measurements of the ESR/FMR spectra close to the coincidence angle with magnified ESR spectral region.

**Figure 3:**
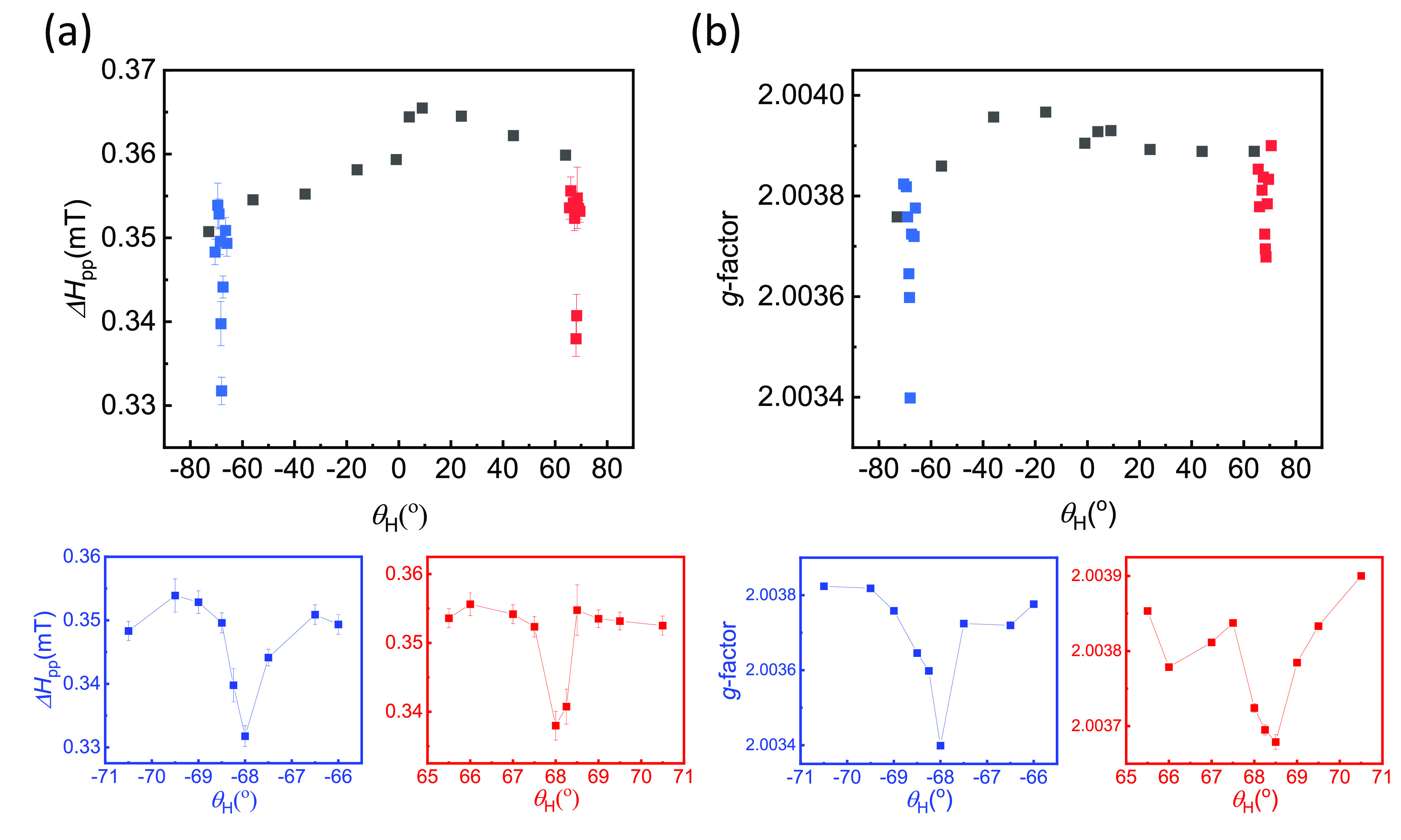
Radical ESR linewidth *ΔH*_pp_ (a) and *g*-factor (b) in the EBR/NiFe bilayer. The bottom row presents the same data for the cases of simultaneous ESR and FMR resonance (*θ*_H_ ≈ ±68°).

The change in the applied magnetic field angle, *θ*_H_, during the resonance measurements causes a variation in the FMR resonance field, *H*_FMR_ (Figure 2b), which is typical for an ultrathin ferromagnetic film.^37^ In contrast, there is no measurable influence on the radical resonance field, *H*_ESR_, as a function of *θ*_H_ (Figure 2b), ruling out the possibility of long-range spontaneous magnetization in the EBR film.^38^ By setting *θ*_H_ = 0° as the in-plane direction, we focus on a range of *θ*_H_, around ±68°, where the resonance of the FMR and the ESR spectra coincide, that is, the two systems can be driven in resonance simultaneously. Detailed measurements of the ESR/FMR spectra close to this coincidence angle are presented in Figure 2c. The *g*-factor and ESR linewidth, *ΔH*_pp_, were determined from the spectra at different *θ*_H_ and are plotted in Figure 3a,b, respectively. A clear reduction of the *g*-factor and the linewidth was observed around the angle where the NiFe and the EBR films were driven in resonance simultaneously. This is more pronounced in an alternative representation of the same data, in Figures S3(b) and S4, where the *g*-factor and *ΔH*_pp_ are plotted as a function of the field separation of the two resonances and a decrease was observed in both when *H*_ESR_ – *H*_FMR_ ≈ 0. A comparison of the ESR linewidth of the bilayer with the single EBR film (Figure 4a) shows an increase in *ΔH*_pp_ for the former for all *θ*_H_ angles except in the regions of simultaneous FMR and ESR (*θ*_H_ ≈ ±68°). In this angle range, *ΔH*_pp_ decreases down to the value of the single radical film. This observation is the most important result of the present study. It clearly demonstrates that the effect observed was dynamic, only occurring when the two resonances were brought together and that it was reversible. This excluded any possibility of a “permanent” increase of the ESR linewidth in the bilayer due to alteration of the radical nature through interaction with the NiFe layer, for example a linewidth increase which could result from a higher intermolecular distance.^36,39^

**Figure 4:**
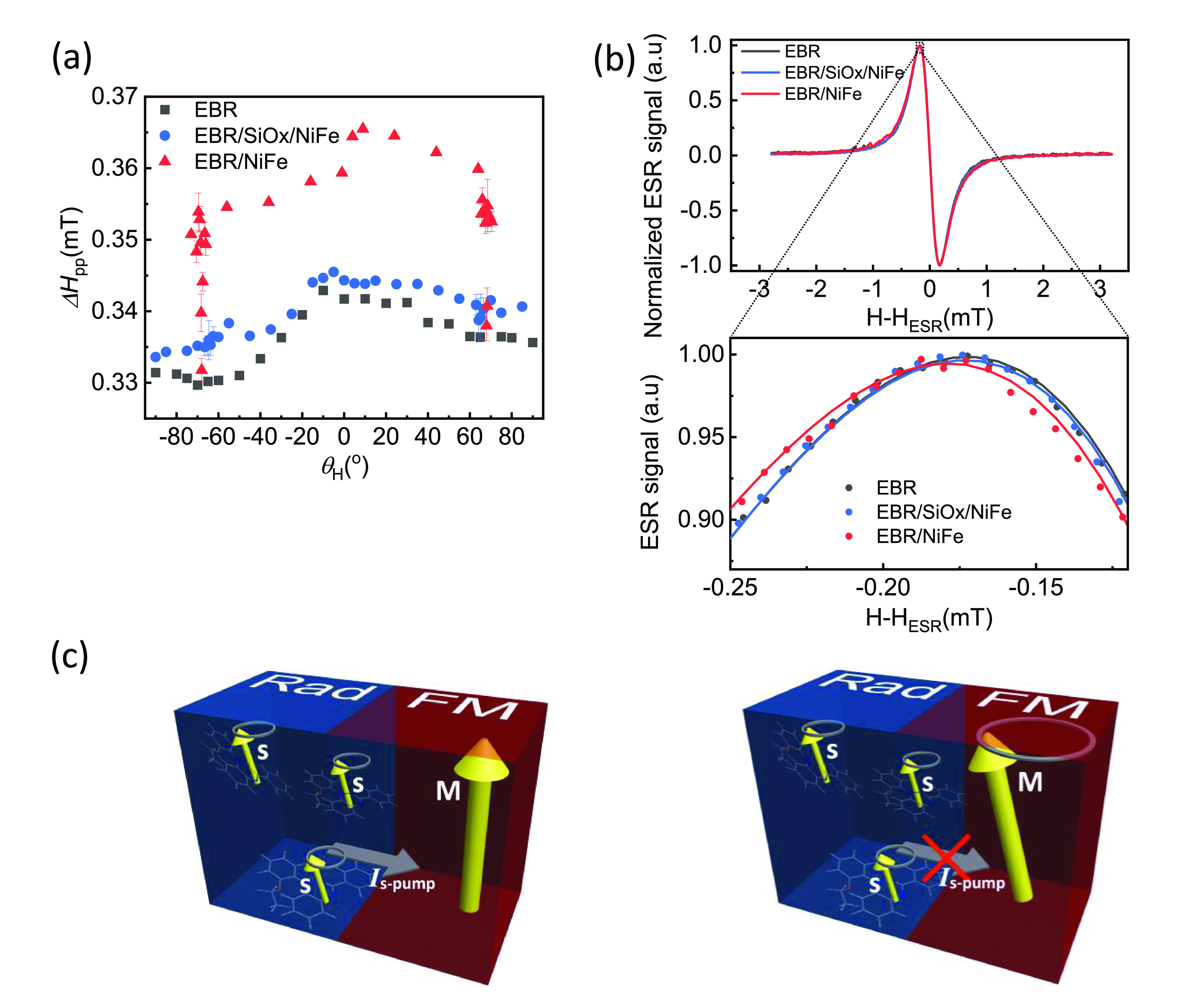
(a) The magnetic field angle, *θ*_H_, dependence of the ESR linewidth, *ΔH*_pp_, for the EBR thin film, the EBR/SiO_x_/NiFe trilayer, and the EBR/NiFe bilayer. (b) Normalized ESR spectra at *θ*_H_ = 0°, of the pristine EBR film, the EBR/SiO_x_/NiFe trilayer, and the EBR/NiFe bilayer. The figure below shows the magnified region of the experimental results around the peak clarifying the shift due to the increase in linewidth, while the solid lines are fits to the data, given by a Lorentzian function. (c) Schematic of spin pumping procedure in a radical/ferromagnet (Rad/FM) bilayer. When the EBR layer is at resonance, it pumps a spin current, *I*_s-pump_, into the NiFe layer, which is detuned from its FMR (left). For simultaneous resonance of the radical and the ferromagnet, the magnitude of *I*_s-pump_ is canceled by an opposite spin current from the FM layer (right).

We attribute the ESR linewidth increase in the bilayer to spin pumping^9,40^ from the radical into the FM, which acts as a perfect spin sink. In general, the term spin pumping refers to the transport of spin angular momentum from a material which experiences magnetic resonance to an adjacent spin sink layer.^40^ Such spin angular momentum transfer causes an increase in magnetization damping, attributed to angular momentum conservation, which is reflected in practice as a broadening of the absorption spectrum, expressed as a linewidth increase.^37,40^ The mechanism of spin pumping has been proposed by Tserkovnyak et al.^40−42^ and is widely used in conducting, e.g., NiFe, Fe,^37,43,44^ and insulating, e.g., YIG, ferromagnetic materials.^45^ More recently, it has been demonstrated in the absence of ferromagnetic order from the paramagnetic insulator La_2_NiMnO_6_.^10^ Conventionally spin pumping from ferromagnetic metals and insulators is described through a macrospin approach using a Landau-Lifshitz-Gilbert (LLG) equation. However, such an approach cannot be applied to paramagnets, such as organic radicals at room temperature,^10,11,13^ since the so-called spin mixing conductance, controlling the spin current through an interface, cannot be defined in the absence of magnetization. A macrospin description can, however, offer a qualitative understanding of the linewidth increase as spin pumping from the radical layer into the FM, which acts as a perfect spin sink. Such a description has previously been useful to describe the spin dynamics for systems with high spin concentration.^46^

The realization of spin angular momentum transfer from the radical film to NiFe requires interaction of the radical’s localized interface spins with the corresponding neighboring NiFe free electrons, which could proceed via interface exchange between the two materials.^10,11,15^ When a magnetic field, *B*, is applied, the energy levels of up- and down-spin states are nondegenerate, differing by the Zeeman energy of *gμ*_B_*B*, where *μ*_B_ is the Bohr magneton. In the presence of interfacial exchange coupling, an up (down) spin itinerant electron in NiFe interacts with a localized down (up) spin in the radical film, causing a spin-flip equivalent to a transfer of spin angular momentum of ±ℏ and energy of ±*gμ*_B_*B*.^11,15^ As a result, the spin relaxation time during ESR is decreased with a corresponding broadening of the spectral linewidth, in a manner that is analogous to the broadening of FMR linewidth in conventional spin pumping from ferromagnets.^37,40^ This increase in the linewidth applies for the whole EBR film, since no deviation from a uniform Lorentzian is observed, indicating a homogeneous broadening system. Spin transport enabling the transfer of angular momentum throughout the EBR could proceed via two mechanisms as in other organic materials. It can be mediated through carrier hopping, which results in simultaneous spin and charge transport,^47,48^ and in an environment with a large spin concentration via exchange coupling between localized spins.^49,50^ In our case, the EBR film exhibits remarkable conductivity σ ≈ 5 × 10^–4^ S cm^–1^ (measurement details in the SI), of the same order with the highest conductivity Blatter radical derivative measured to date,^25^ indicating the possibility of hopping transport. This is in contrast to most neutral π radicals, which are insulators or poorly conducting semiconductors with σ < 10^–10^ S cm^–1^.^51^ Furthermore, using the doubly integrated ESR spectra of the EBR calibrated against the standard DPPH *S* = 1/2 radical, we obtain the spin density in the EBR film and, consequently, a mean distance between the radical spin centers of ∼0.4 nm, for which a strong exchange interaction is expected.^30,31^ This is consistent with the ESR spectrum having a single Lorentzian shape, a feature characteristic of high spin density radical systems with strong exchange between neighboring molecules.^52,53^ Therefore, a contribution to spin transport within the radical film from both mechanisms cannot be ruled out.

A contribution to the observed increase of *ΔH*_pp_ can also arise from factors unrelated to spin pumping, for example, due to a permanent alteration of the nature of the radical molecule itself when deposited on a metallic surface, resulting in the increase of *ΔH*_pp_.^27,39^ This possibility, however, can be ruled out by careful consideration of the linewidth measurements of Figure 4a for simultaneous FMR and ESR, where the increase of the linewidth is quenched and *ΔH*_pp_ reduces to the value of the single radical layer. This observation is inextricably linked with spin pumping. Analogous linewidth reduction due to spin pumping was previously observed for FM_1_/normal metal (NM)/FM_2_ trilayers when the resonant fields of two ferromagnetic layers coincide.^54^ More thorough investigations of this effect followed, both theoretically^55^ and experimentally for trilayers FM_1_/NM/FM_2_ or bilayers FM_1_/FM_2_ for the same^56,57^ and for different FMs.^58−60^ More recently, the magnetization coupling in a paramagnetic/FM bilayer has also been reported.^61^ The simultaneous resonance of the two FM layers results in coherent precession and simultaneous pumping of spin currents in opposite directions from one layer into the other. The case where the precession of the two layers is in-phase results in no linewidth increase; effectively, the spin current emitted by one layer is matched by equivalent absorption of spin current received from the other FM layer.^54,55^ The observed vanishing of the increase in *ΔH*_pp_ can be attributed to an in-phase coherent precession of the radical and the NiFe (Figure 4c) and a consequent emission of counter-propagating in-phase spin currents. When the EBR film is in resonance alone, it emits a spin current via spin pumping into the NiFe, and its linewidth is enhanced (Figure 4c, left). For simultaneous resonance, the EBR film emits and simultaneously receives the spin current emitted by the NiFe layer through their interface. The former is causing an increase of *ΔH*_pp_ while the latter a decrease, eliminating the enhancement in the linewidth due to spin pumping (Figure 4c, right). In order to support the explanation of spin pumping, a control measurement was carried out by inserting a SiO_x_ layer between the EBR and the NiFe layer, to act as a spin blocker. The results in Figure 4a show no enhancement in the linewidth when the ESR and FMR resonances do not coincide. Furthermore, there is no linewidth decrease when the two resonances coincide. Figure 4b shows typical ESR spectra of the EBR, EBR/SiO_x_/NiFe, and EBR/NiFe samples. The linewidth of the latter is clearly different from the others as expected for the organic radical acting as spin source into the NiFe spin sink.

Another consequence of the simultaneous ESR and FMR resonance is the observed decrease of the *g*-factor (Figure S3) associated with a corresponding shift of the resonance field in the ESR spectra. A similar shift in the resonance field near simultaneous resonance is reported for coupled ferromagnetic bilayers^58,60^ due to a fieldlike torque acting along or against the Larmor precession, with possible contributions from both interfacial exchange coupling^58,60^ and spin current transport.^60^ Accordingly, the *g*-factor decrease at *θ*_H_ ≈ ±68° can be qualitatively understood in the context of a similar macrospin model where the incoming spin current from the NiFe layer can cause the resulting field-like term, which acts as an effective field or equivalently a shift in the *g*-factor.

Τhe successful emission of spin current from the EBR indicates the possibility of realizing spin pumping even in the absence of long-range ferromagnetic order. Further evidence toward this possibility is the successful observation of spin pumping from a crystalline inorganic paramagnet, showing the same relative change in linewidth (∼10%) as in the present study,^10^ together with a theoretical description for spin pumping from a fluctuating ferromagnet near *T*_c_.^13^ These reports and the results presented here suggest that a generalization of the spin pumping theory is required beyond systems with long-range ferromagnetic order and in particular for the case of organic radicals.

In conclusion, we have demonstrated spin current creation via spin pumping from a purely organic radical following the successful fabrication of stable radical films at room temperature. The spin resonance linewidth and the *g*-factor can be dynamically controlled via absorption by the radical film of a spin current emitted from a nearby ferromagnet. The present study illustrates the potential of organic radicals to act as spin sources in future spintronic devices.

## Supporting Information Available

Synthesis and characterization of the Blatter radical derivatives with additional supporting results including AFM and conductivity measurement and a detailed presentation of ESR fitting procedure (PDF)
