# Supplementary material for: Metal-Free Organic Radical Spin Source
Source: Nano Lett. 2023 May 8;23(10):4579–86. doi: 10.1021/acs.nanolett.3c01044 (PMC10214490; doi:10.1021/acs.nanolett.3c01044)
Supplement: Supplementary file 1 — nl3c01044_si_001.pdf [file nl3c01044_si_001.pdf]

SUPPORTING INFORMATION

Metal-free Organic Radical Spin Source

Constantinos Nicolaides,[1] *Fadwat Bazzi,*[2] *Evangelos Vouros,*[1] *Dragos F. Flesariu,*[3] *Nicolas Chrysochos,*[3] *Panayiotis A. Koutentis,*[3] *Christos P. Constantinides,*[2] *Theodossis Trypiniotis*[1]*

[1] Department of Physics, University of Cyprus, P.O. Box 20537, 1678 Nicosia, Cyprus
[2] Department of Natural Sciences, University of Michigan – Dearborn, 4901 Evergreen Rd, Dearborn, MI 48128-1491, United States
[3] Department of Chemistry, University of Cyprus, P.O. Box 20537, 1678 Nicosia, Cyprus

Contents Page

Experimental Details 2

Cyclic Voltammetry 4

ESR spectroscopy 5

UV/vis & FTIR-ATR spectrum of EBR 7

AFM Measurement 8

ESR fitting procedure 9

Conductivity Measurement 10

Experimental Details

General Methods and Materials. All chemicals were commercially sourced, except those whose synthesis is described. CH_2Cl_2 and THF were freshly distilled from CaH_2 under argon. Reactions were protected from atmospheric moisture by $CaCl_2$ drying tubes. All reaction mixtures and column eluents were monitored by thin-layer chromatography (TLC) using commercial aluminum-backed TLC plates (Merck Kieselgel 60 F_{254}) TLC plates were observed under UV light at 254 and 365 nm. The technique of dry flash chromatography[1] was used throughout for all non-TLC-scale chromatographic separations and employed silica gel 60 (< 0.063 mm). Melting and decomposition points were determined using either a PolyTherm-A, Wagner & Munz, Koefler–Hostage Microscope apparatus. The solvent used for recrystallization is indicated after each melting point. UV/vis spectra were obtained using a PerkinElmer Lambda-25 UV/vis spectrophotometer. IR spectra were recorded on Shimadzu FTIR-NIR Prestige-21 spectrometer with a Pike Miracle Ge ATR accessory; strong, medium, and weak peaks are represented by “s”, “m”, and “w”, respectively. MALDI-TOF mass spectra were recorded on a Bruker Autoflex III Smartbeam instrument. Elemental analysis was performed on a PerkinElmer 2400 series elemental analyzer at London Metropolitan University. 3-Phenylbenzo[*e*][1,2,4]triazine was prepared according to the literature.[2]

The $Ni_{81}Fe_{19}$ ferromagnetic thin deposition of 7nm thickness, was deposited on a Si/SiO_x substrate at room temperature in molecular beam epitaxy system by e-beam evaporation. The deposition rate was monitored by a water-cooled quartz microbalance and was constant around 0.32 Å/min. The base and growth pressure were $\sim 9\times10^{-9}$ mbar and 3×10^{-8} mbar, respectively.

The synthesis of 1-(2-ethoxyphenyl)-3-phenyl-1,4-dihydro-1,2,4-benzotriazin-4-yl (EBR) followed the same protocol as MBR, *i.e.*, addition of 2-lithiophenetole to 3-phenylbenzo[*e*]-[1,2,4]triazine in dry THF at -78 °C under an argon atmosphere (Scheme S1).[3] Generation of 2-lithiophenetole was carried out *via* the directed ortho lithiation of phenetole. We established that the complete consumption of 3-phenylbenzo[*e*][1,2,4]triazine requires 2 equivalents of 2-lithiophenetole. The dark solution of the resulting anion was exposed to air and left stirring overnight to oxidize and generate EBR which was purified by column chromatography and recrystallized from *c*-hexane to afford black prisms.

Scheme S1. Synthetic route to EBR.

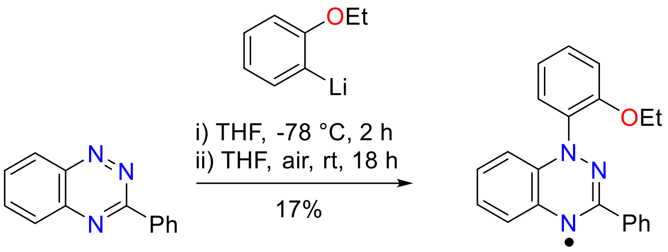

1-(2-Ethoxyphenyl)-3-phenyl-1,4-dihydrobenzo[*e*][1,2,4]triazin-4-yl (EBR). To a stirred solution of phenetole (0.25 mL, 2.00 mmol) in anhydrous THF (5 mL) at *ca.* 0 °C (ice bath) under an Ar atmosphere, was added dropwise a 1.6 M solution of *n*-butyllithium in hexanes (1.30 mL, 2.10 mmol). The solution was left to stir at *ca.* 0 °C for 1 h and then allowed to warm to *ca.* 20 °C for 2 h. A separate flask was charged with a solution of 3-phenylbenzo[*e*][1,2,4]triazine (207 mg, 1.00 mmol) in anhydrous THF (5 mL) and was cooled to *ca.* -78 °C (dry ice/acetone). To this cooled benzotriazine solution at *ca.* -78 °C under an Ar atmosphere was added dropwise the prepared solution of (2-ethoxyphenyl)lithium. The mixture was left to stir at *ca.* -78 °C for 1 h, then allowed to warm to *ca.* 20 °C and stirred for an additional 1 h. The reaction was opened to air and left to stir overnight (~12 h). Volatiles were then removed *in vacuo* and the residue obtained was dissolved in CH_2Cl_2 (40 mL), washed (water, 3 × 50 mL), dried ($MgSO_4$), chromatographed and recrystallized (117 mg, 17%) as black prisms; mp (hot-stage) 108-110 °C (*n*-hexane); R_f 0.48 (*n*-Hex/*t*-BuOMe, 80:20); Anal. Calcd. for $C_{21}H_{18}N_3O$: C, 76.81; H, 5.53; N, 12.80. Found C, 77.01; H, 5.43; N, 13.14%; λ_{max} (CH_2Cl_2)/nm 270.5 (log ε 4.59), 329 (3.58), 367 (3.80), 414 (3.42), 491 (3.12); ν_{max}/cm^{-1} 3057w, 3032w, 2981w, 1597m, 1578w, 1559w, 1539w, 1501s, 1483s, 1450m, 1391s, 1331m, 1317m, 1308w, 1283s, 1273m, 1250m, 1238m, 1207m, 1176w, 1163m, 1148w, 1128m, 1113m, 1088w, 1042s, 986w, 959w, 920m, 894.97w, 878w, 858w, 845w, 824w, 818w, 779m, 760s, 746m, 727w, 717w; *m*/*z* (MALDI) 329 (MH^+ 24%), 328 (M^+ 100); g = 2.00402 (CH_2Cl_2, 20 °C).

Cyclic Voltammetry. Studies were performed on a Princeton Applied Research Potentiostat / Galvanostat 263A. The concentrations of 1-(2-ethoxyphenyl)-3-phenyl-1,4-dihydrobenzo[*e*]-[1,2,4]triazin-4-yl (EBR) was 1 mM in CH_2Cl_2 containing *n*-Bu_4NPF_6 (0.1 M) as an electrolyte. A three-electrode electrochemical cell was used with glassy carbon disk as working electrode, Pt wire as counter electron and Ag/AgCl (1 M KCl) as reference electrode. Scan rate 100 mV s^{-1}. Temperature = 20 °C. Fc/Fc^+ (E_{Fc/Fc^+} = 0.46 V *vs* SCE) was used as an internal reference.[4] CH_2Cl_2 was distilled over CaH_2. Samples were deaerated by passing argon through the solvent, prior to measuring the cyclic voltammogram. For all measurements, blank samples (only electrolyte in the system) were taken to ensure the correct operation of the electrochemical cell. Upon each measurement, ferrocene was added and the cyclic voltammogram was taken again. All redox potentials ($E_{1/2}$) are referenced according to the ferrocene's value ($E_{Fc/Fc^+} = 0.46\ V$ *vs* SCE).[5]

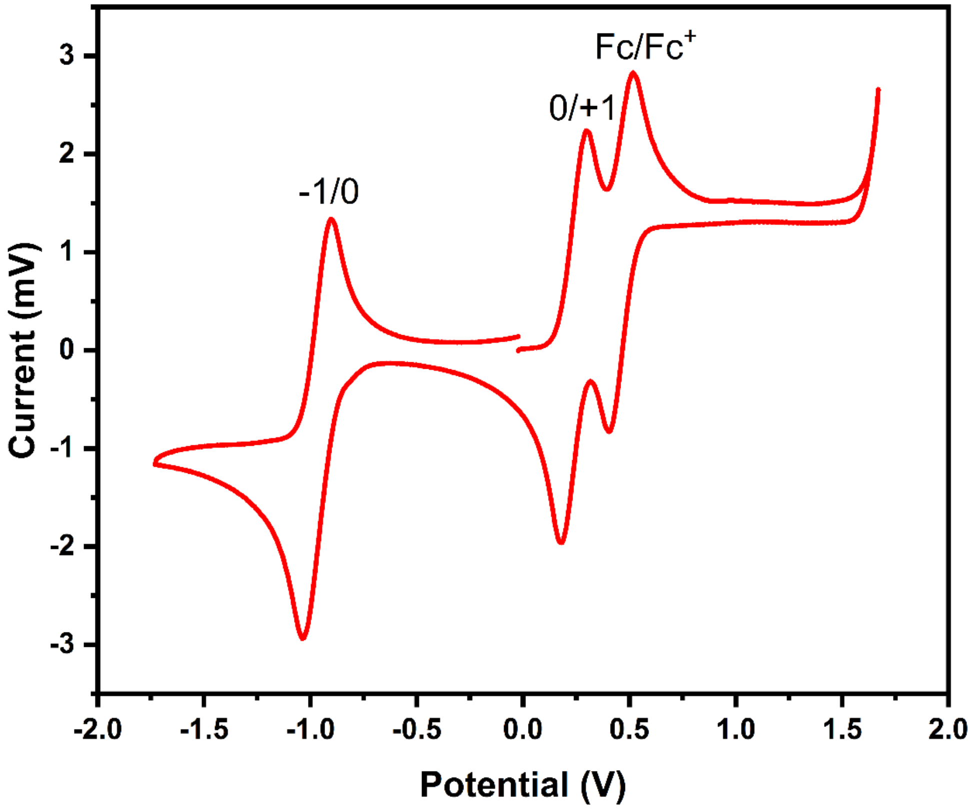

Figure S1. CV data of 1-(2-ethoxyphenyl)-3-phenyl-1,4-dihydrobenzo[*e*][1,2,4]triazin-4-yl (EBR): $E_{1/2}^{0/+1}$ = 0.237 V, $E_{1/2}^{-1/0}$ = -0.970 V, E_{cell} = 1.207 V.

ESR Spectroscopy. ESR spectra of EBR (1 mM solution in CH_2Cl_2) were recorded on an X-band ESR spectrometer at 20 °C. Simulations of the solution spectra were performed using EasySpin.[6]

(a)

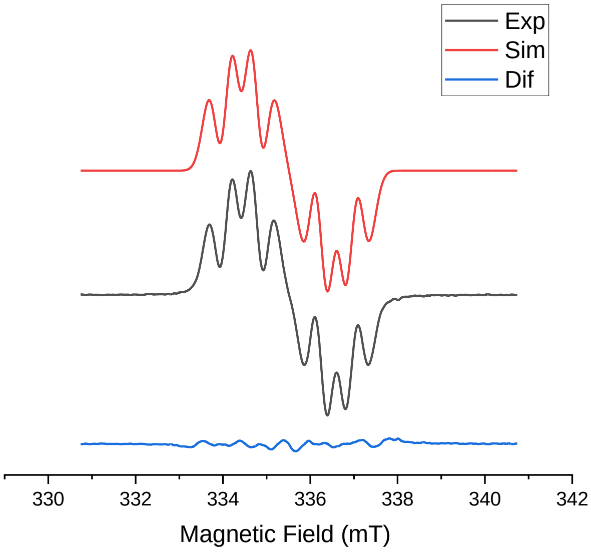

g-factor = 2.00402, aN/G = 7.251, aN/G = 4.555, aN/G = 4.684, ΔHpp/G = 3.96.

(b)

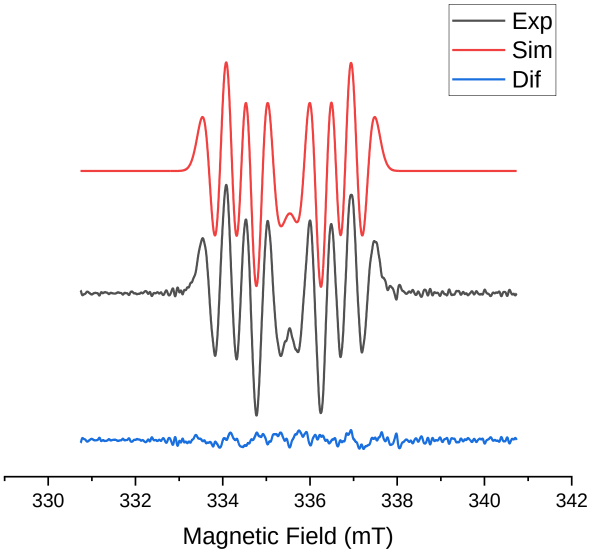

g-factor = 2.00403, aN/G = 7.249, aN/G = 4.453, aN/G = 4.767, ΔHpp/G = 3.839.

Figure S2. Experimental (CH_2Cl_2, *ca*. 20 °C) and simulated first (a) and second (b) order ESR spectra of ESR along with their simulation parameters.

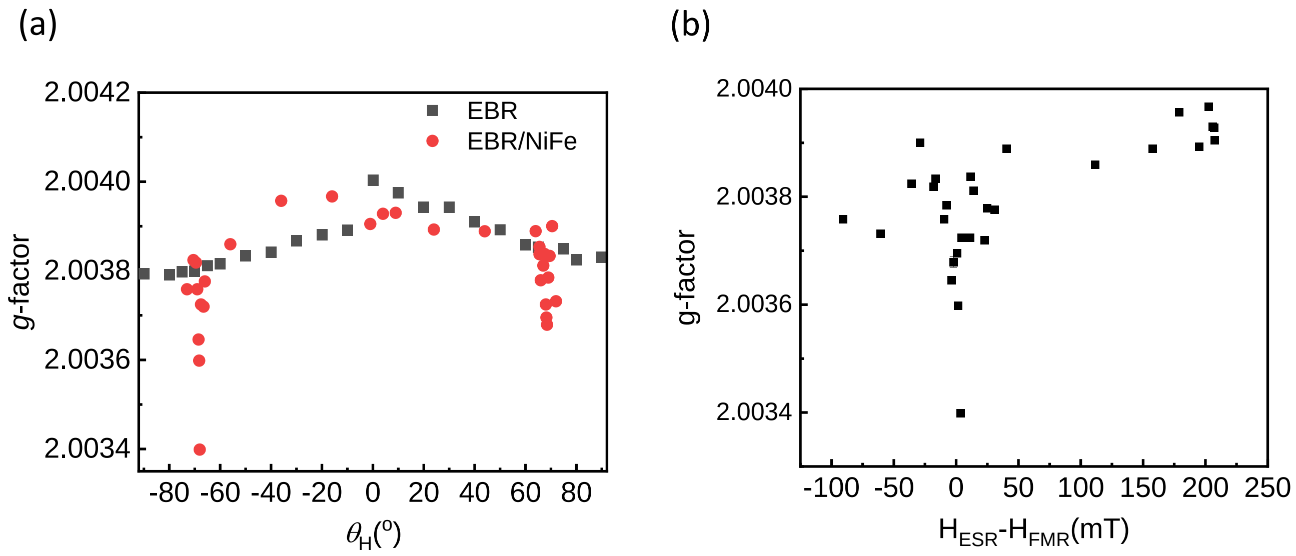

Figure S3. The *g*-factor of the EBR/NiFe bilayer and the EBR/SiOx/NiFe trilayer as a function of as a function of θ_H angle (b) as a function of the field separation of the two resonances, H_{ESR}- H_{FMR}.

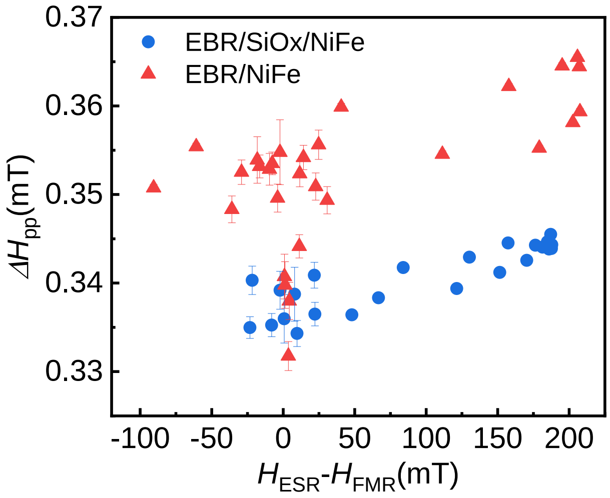

Figure S4. The linewidth, ΔH_{pp}, of the EBR/NiFe bilayer and the EBR/SiOx/NiFe trilayer as a function of the field separation of the two resonances, H_{ESR}- H_{FMR}.

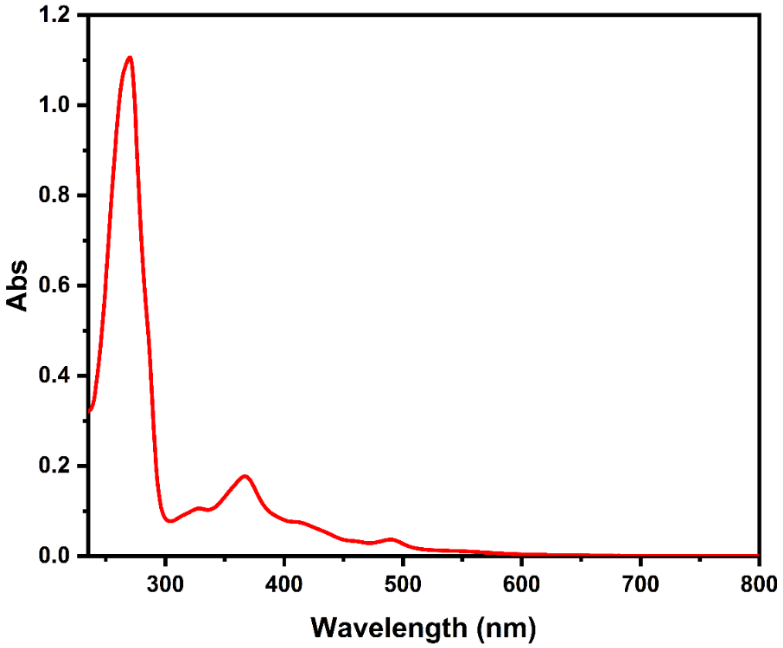

Figure S5. UV/vis spectrum of EBR at 20 °C measured at *ca.* 2.7 × 10^{-5} M.

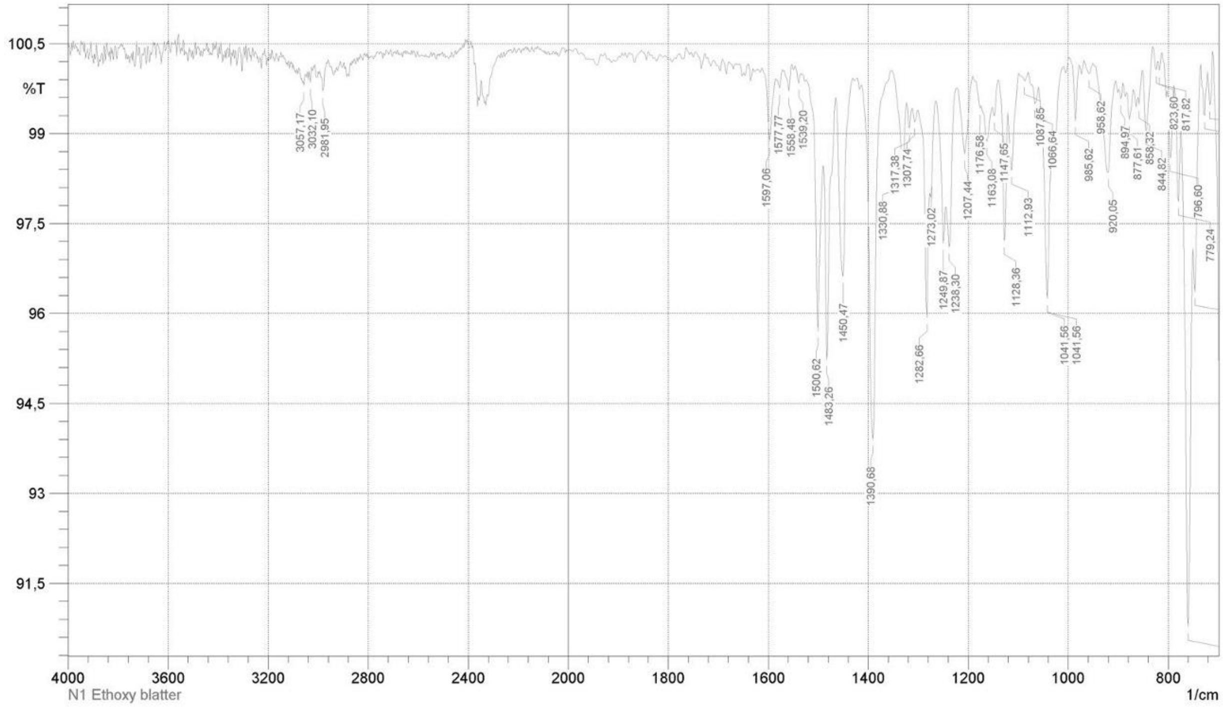

Figure S6. FTIR-ATR spectrum of EBR.

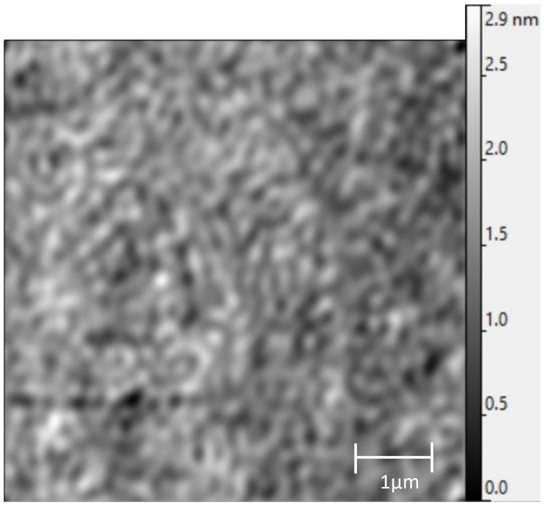

Figure S7. A typical 6 μm × 6 μm AFM image from which a root mean square (rms) roughness 250 ± 30 pm was calculated.

ESR fitting procedure

(a) EBR/NiFe bilayer at $\theta_{\mathrm{H}} = 0^{\mathrm{o}}$

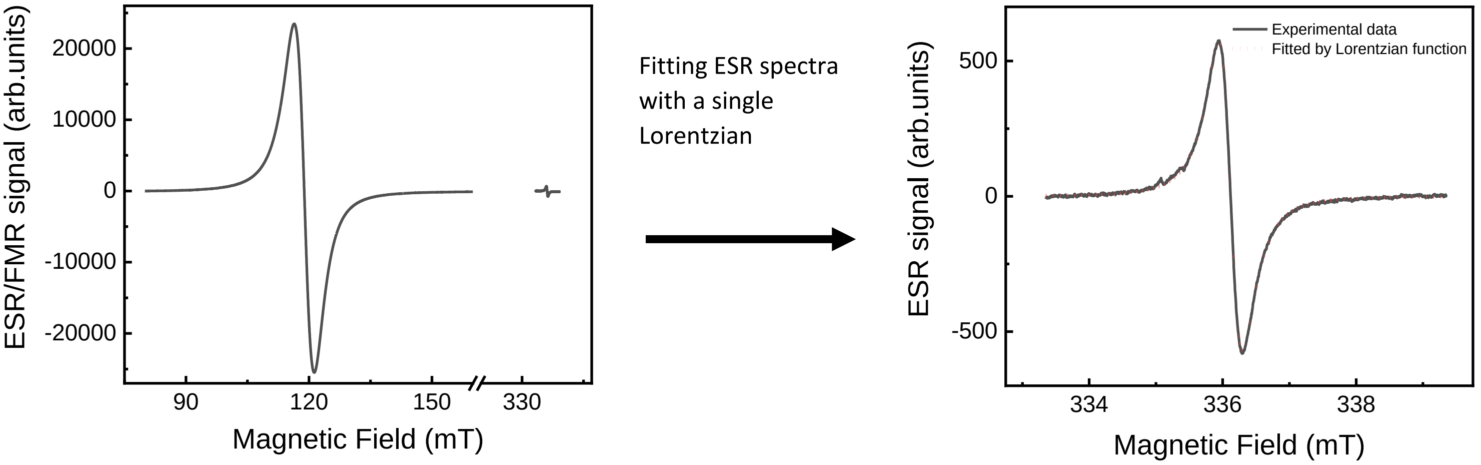

(b) EBR/NiFe bilayer at $\theta_{\mathrm{H}} = 68.25^{\mathrm{o}}$

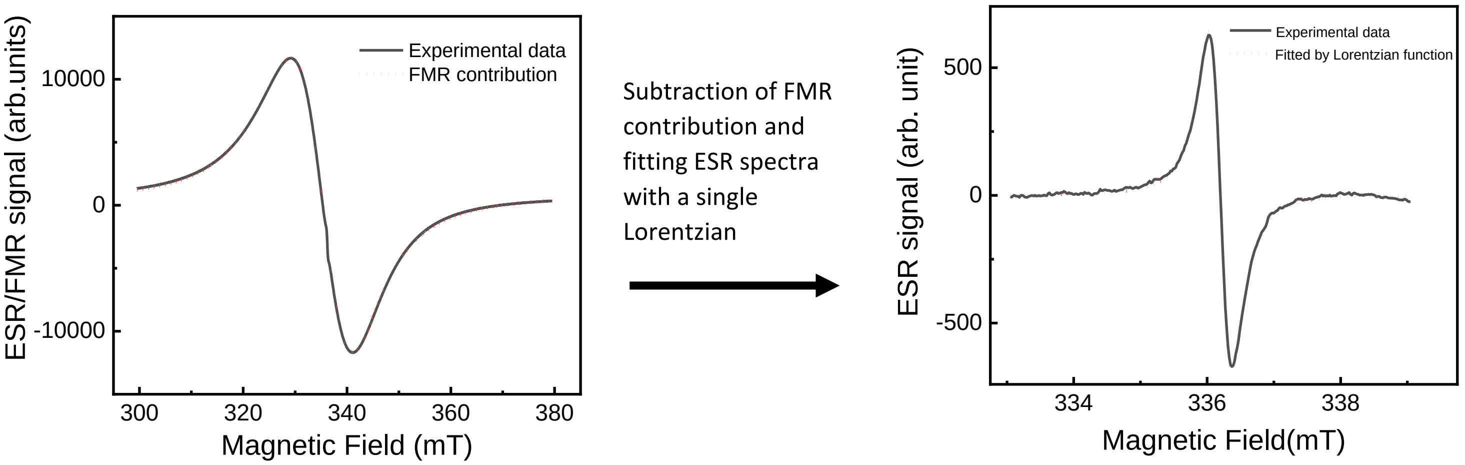

(c) EBR/NiFe bilayer at $\theta_{\mathrm{H}} = 69^{\mathrm{o}}$

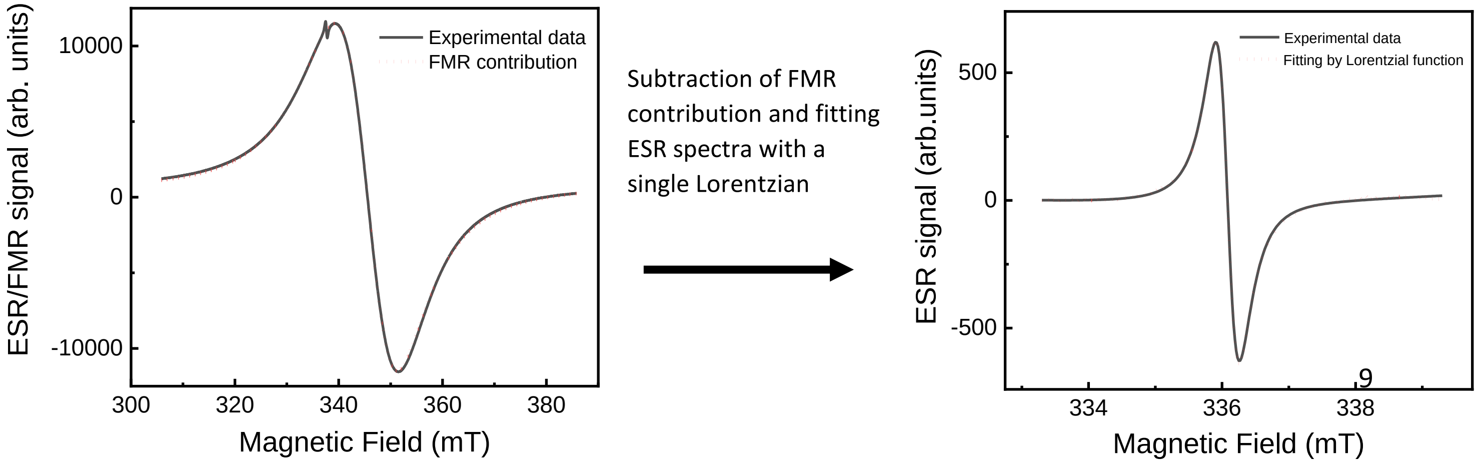

(d) EBR thin film monolayer at $\theta_H = 0^o$

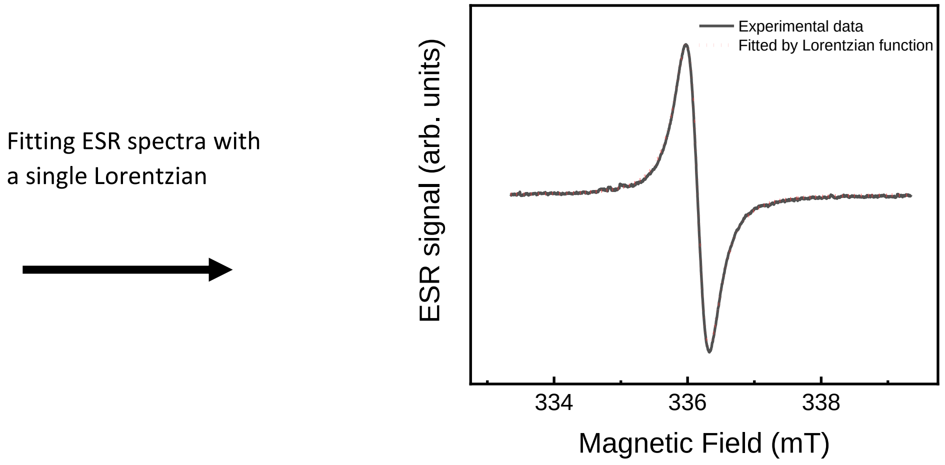

Figure S8. Detailed presentation of the fitting procedure of ESR spectra for the EBR/NiFe bilayer at (a) $\theta_H = 0°$, (b) $\theta_H = 68.25°$, (c) $\theta_H = 69°$ and the EBR single layer (d) at $\theta_H = 0°$.

Conductivity measurement. The conductivity of EBR thin films was measured using two-probe method. Two Au electrodes with a spacing of 40 μm [Figure S8(a)] were thermally evaporated on the film and were connected to Keithley 2450 sourcemeter. The IV curve was measured and combined with the thickness obtained by AFM the conductivity was estimated $\sigma \approx 5 \times 10^{-4}$ Scm^{-1}.

(a)

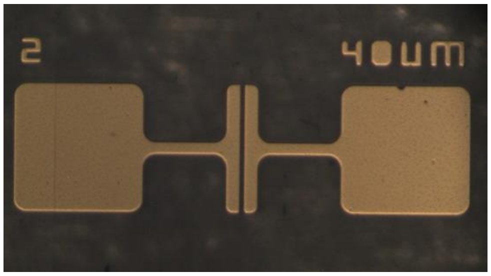

(b)

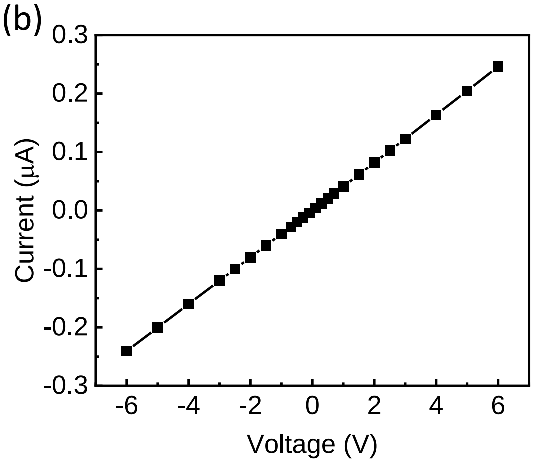

Figure S9. (a) Device used for the measurement of EBR film's conductivity. (b) Typical I-V curve of an EBR film.

References

1. Hardwood, L. M. Dry-Column Flash Chromatography. *Aldrichimica Acta* **1985**, *18*, 25.
2. Constantinides, C. P.; Obijalska, E.; Kaszynski, P. Access to 1,4-Dihydrobenzo[*e*][1,2,4]triazin-4-yl Derivatives. *Org. Lett.* **2016**, *18*, 916-919.
3. Bazzi, F.; Danke, A. J.; Lawson, D. B.; Manoli, M.; Leitus, G. M.; Koutentis, P. A.; Constantinides, C. P. 1-(2-Methoxyphenyl)-3-phenyl-1,4-dihydro-1,2,4-benzotriazin-4-yl: a tricky "structure-to-magnetism" correlation aided by DFT calculations, *CrystEngComm* **2020**, *22*, 4306-4316.
4. Shirley, D. A.; Johnson, J. R.; Hendrix, J. P. *J. Organomet. Chem*. **1968**, *11*, 209-216.
5. Connelly, N. G.; Geiger, W. E. Chemical Redox Agents for Organometallic Chemistry. *Chem. Rev.* **1996**, *96*, 877-910.
6. Stoll, S.; Schweiger, A. EasySpin, a comprehensive software package for spectral simulation and analysis in EPR. *J. Magn. Reson.* **2006**, *178*, 42-55.
